# Retraction Notice to: Inflammatory-Related P62 Triggers Malignant Transformation of Mesenchymal Stem Cells through the Cascade of CUDR-CTCF-IGFII-RAS Signaling

**DOI:** 10.1016/j.omtn.2022.12.003

**Published:** 2022-12-11

**Authors:** Xiaoru Xin, Chen Wang, Zhuojia Lin, Jie Xu, Yanan Lu, Qiuyu Meng, Xiaonan Li, Yuxin Yang, Qidi Zheng, Xin Gui, Tianming Li, Hu Pu, Wujun Xiong, Jiao Li, Song Jia, Dongdong Lu

## Main text

(Molecular Therapy: Nucleic Acids *11*, 367-381; June 2018)

This article has been retracted at the request of the editor-in-chief. Similarities were found between images in this article and an article in *Cell Death and Disease* (Zheng et al., 2018, Cell Death Dis. *15*, 253, https://doi.org/10.1038/s41419-018-0305-7). All authors of the *Cell Death and Disease* article appeared as authors of the *Molecular Therapy - Nucleic Acids* article. Image analysis performed by the editorial office confirmed findings of image duplication in Figure 4B of the *Molecular Therapy - Nucleic Acids* article. This reuse (and in part misrepresentation) of data without appropriate attribution represents a severe abuse of the scientific publishing system.

